# Comparison of Nonimage- and Fluoroscopy-Guided Interlaminar Epidural Block: A Matched Paired Analysis in the Same Individuals

**DOI:** 10.1155/2019/7513617

**Published:** 2019-04-01

**Authors:** Syn-Hae Yoon, Hanwool Park, Kunhee Lee, Haesol Han, Keum Nae Kang, Gunn Lee, Yun A Han, Seong-Soo Choi

**Affiliations:** ^1^Department of Anesthesiology and Pain Medicine, National Police Hospital, Seoul, Republic of Korea; ^2^Department of Anesthesiology and Pain Medicine, Asan Medical Center, University of Ulsan College of Medicine, Seoul, Republic of Korea

## Abstract

**Background:**

Although fluoroscopic guidance is recommended highly for more accurate lumbar interlaminar epidural steroid injection (L-ESI), many physicians still use a nonimage-guided approach for L-ESIs. However, because of its associated risk of radiation and increased medical expense, the cost-effectiveness and safety of fluoroscopy-guided ESI have been called into question. The goal of this retrospective matched paired analysis in the same individuals was to assess the effectiveness and prevalence of complications of nonimage-guided L-ESI compared to those of fluoroscopy-guided L-ESI*. Methods*. Between 2015 and 2016, 94 patients who received both nonimage- and fluoroscopy-guided L-ESIs were analyzed retrospectively. The changes of the numeric rating scale (NRS) in pain intensity and functional outcome and the differences in the number of complications between blind and fluoroscopy-guided L-ESIs in the same individuals were evaluated by a matched paired analysis.

**Results:**

Of the 94 patients, the differences in NRS before and after the procedure were 1.29 (95% confidence interval (CI) = 0.94–1.65) for the nonimage-guided group and 1.64 (95% CI = 1.28–2.01) for the fluoroscopy-guided group (*p*=0.16). More subjective functional improvement was observed in fluoroscopy-guided L-ESI (57, 60.6%) than in nonimage-guided L-ESI (47, 50.0%) without statistical significance (*p*=0.16). Nine (9.6%) patients in the nonimage-guided group experienced complications related to the procedure overall compared to 4 (4.3%) in the fluoroscopy-guided group (*p*=0.27).

**Conclusions:**

In this study, both blind and image-guided L-ESI techniques included similar extents of postprocedural outcomes and complications. Physicians should consider the risks associated with the two different techniques overall and develop ways to individualize the procedure to decrease the risk of complications and improve the positive outcomes of lumbar epidural steroid injections.

## 1. Introduction

Traditionally, lumbar interlaminar epidural steroid injections (L-ESIs) have been performed without radiographic guidance. Over the past two decades, image-guided fluoroscopy has been adopted in practice and rapidly has become available to achieve more accurate procedures [1]. Most of the studies of the effectiveness of the L-ESIs had low success rates of pain relief when were performed without image guidance [2]. Nevertheless, many physicians still use a nonimage-guided technical approach that relies largely on the loss of resistance technique [2, 3]. Because patient response depends on accurate placement of the drugs injected, it is important to determine the effectiveness of nonimage-guided L-ESIs because of the questionable evidence of this procedure's accuracy when performed blindly.

Because of the radiation hazard and increased medical expense, the cost-effectiveness and safety of fluoroscopy-guided L-ESIs have been called into question. Both patients and operators are at significantly increased radiation risk because of the widespread use of fluoroscopy-guided interventional procedures [4, 5]. Although the risks of chronic occupational exposure to low level radiation are less clear [4], the risks of radiation to the fetus and risk of cataracts in interventional radiologists have been established relatively more clearly [6]. In addition to radiation risk, the medical expense associated with the fluoroscopy-guided procedure also should be justified, as the one-step course of blind L-ESIs is performed more frequently with less expensive resources, particularly in outpatient private pain clinics [1, 7].

Because of the discrepancy between the technically ideal approach and the approach used more frequently, this retrospective analysis was undertaken to assess data on the effectiveness, rate of failure, and prevalence of iatrogenic complications of nonimage-guided, blind L-ESI compared to those of fluoroscopy-guided L-ESI with a focus on lower back pain in the same patients. The retrospective review of the patients who went through both fluoroscopy-guided and nonimage-guided L-ESI were analyzed. This study was the first to compare the efficacy of both blind and image-guided ESI technique in the same patients. Comparing the two different procedures in the same patients provided us with extremely helpful information because they were performed under exactly the same anatomical conditions.

## 2. Materials and Methods

### 2.1. Patients

This retrospective study was performed at the Pain Clinic in Asan Medical Center, and the protocol was approved by its institutional review board (approval number 2017-0541). We searched our institution's Information Technology of Service Management (ITSM) system between January 2015 and December 2016 using the procedure name, “lumbar epidural steroid injection.” Regardless of whether the procedure was performed with fluoroscopy, both were included in the study if they met the following criteria: (1) patients were at least 20 years of age; (2) patients had chronic lumbar pain that did not respond to conservative therapy, including medical or physical therapy; and (3) patients had undergone both fluoroscopy and nonimage-guided L-ESI. Patients with the following conditions were excluded from the study: (1) uncontrolled psychiatric or medical conditions; (2) experience with fluoroscopy- or nonimage-guided L-ESI alone; (3) lost follow-up from our pain clinic; (4) allergic to local anesthetics, contrast dye, or steroids; and (5) coagulopathy.

According to the retrospective review, reasons to go through the blind technique included (1) nonavailability to use the fluoroscopy on patients visiting days and (2) refusal to go into the OR room or to wait for days to get the procedure. On the other hand, reasons to go through the fluoroscopy-guided technique included the following: (1) patients have enough time to wait until the scheduled operation and (2) patients readily available fluoroscopy on the patients' visiting days.

### 2.2. Procedures

All procedures were performed on an outpatient basis. Nonimage-guided L-ESIs were performed at the outpatient clinic, and fluoroscopy-guided L-ESIs were performed in the operating room. The level of pathology was determined by clinical symptoms and signs, and sensory deficits were correlated with the imaging tests. For the blind L-ESIs, operators used the line connecting the superior iliac crests to estimate the level of the L4 vertebra or the L5-S1 intervertebral space as an anatomical landmark to locate the intervertebral level desired. These anatomical landmarks were corrected and confirmed by simple lumbar spine radiography obtained previously. The patient was placed in the lateral decubitus position with flexion of the knee and hip before the nonimage-guided L-ESI was performed. After sterile skin preparation, infiltration of 1% lidocaine into the skin and subcutaneous tissue was performed in an aseptic fashion. At the interspace closest to the clinical level of pathology, a 20-gauge Tuohy needle (B. Braun, Melsungen, Germany) was placed and the loss of resistance technique was used to guide the needle tip to the epidural space accurately. After confirming no aspiration of blood and/or cerebrospinal fluid, 6 mL of a mixture of 1% lidocaine, 5 mg of dexamethasone, and 1500 IU of hyaluronidase was administered.

In the case of the fluoroscopy-guided L-ESIs, a fluoroscope was used to visualize the needle during the procedure. To monitor incidental neural damage and allow the patients to cooperate during the procedure, medications or sedatives were not used prior to the procedure. The patient was placed on the table in a prone position with a pillow under the abdomen to minimize lumbar lordosis. After sterile preparation of skin and anesthetization of skin and soft tissue with 1% lidocaine, a 20-gauge Tuohy needle was inserted into the target intervertebral space. An epidurogram of the target vertebral level was obtained using a contrast medium (Omnipaue, Nycomed Imaging, Oslo, Norway), and 6 mL of a mixture of 1% lidocaine, 5 mg of dexamethasone, and 1500 IU of hyaluronidase was injected into the fluoroscopy-confirmed epidural space.

In cases of accidental dural puncture, intrathecal needle insertion, motor weakness, sudden hypotension during the procedure, and intravascular drug injection, the needle was removed immediately and the events were noted in the electrical medical records. The epidural injection was attempted at either one intervertebral space above or below the initial attempt when accidental dural puncture occurred.

### 2.3. Outcome Evaluation

The outcome evaluation was performed at baseline and at the next follow up visit after the procedure. For the outcome assessment, the numeric rating scale (NRS) of pain intensity and improvement in physical functional status were reviewed from each patient's medical record. Patients were considered to have better functional outcomes if they said “better” after the procedure. If they expressed “no change or worse” or did not mention the functional change after procedure, the patients were considered no change or worse functional outcomes.

Baseline characteristics, including age, gender, body mass index, underlying diseases, duration of pain, pain intensity, etiology of lumbar pain, and formal reading of diagnostic imaging tests, including magnetic resonance imaging (MRI), lumbar X-rays, and lumbar computer tomographic (CT) tests, were obtained for analysis. We reviewed the lumbar MRIs to determine the principal pathology of lumbar pain and divided our findings into 5 groups: (1) herniated intervertebral disc; (2) foraminal spinal stenosis; (3) central spinal stenosis; (4) failed back surgery syndrome, and (5) facet joint syndrome. Complications associated with the procedure with respect to postdural puncture headache, intrathecal needle insertion, motor weakness, hypotension, and intravascular drug injection also were reviewed in the analysis.

### 2.4. Statistical Analysis

Continuous variables were expressed as means and 95% confidence intervals (CI), and categorical variables were expressed as frequencies or percentages. Continuous variables were compared using a paired *t*-test or Wilcoxon rank sum test to assess differences between the nonimage guided and fluoroscopy-guided L-ESI groups, as appropriate. Categorical data were compared with a McNemar's test to assess differences between the two groups. The data were analyzed using SPSS v. 21.0 (IBM Corp., Armonk, NY).

## 3. Results

A total of 131 charts were reviewed. Of these, 37 were excluded for the following reasons: follow up loss (*n*=12); transforaminal approach to the epidural space (*n*=14); caudal approach to the epidural space (*n*=9), and ESIs performed at the intervertebral space between the 12th thoracic and 1st lumbar vertebra (*n*=2). Ultimately, data from 94 ESIs were analyzed. Of these, patient demographics and diagnosis of lumbar pain are shown in Table 1.

The initial and postprocedure pain intensity between the blind and fluoroscopy-guided interlaminar epidural steroid injections in the same individuals are shown in Table 2. Of the 94 patients, differences in NRS between pre- and postprocedure were 1.29 (95% CI = 0.94–1.65) for the nonimage-guided group and 1.64 (95% CI = 1.28–2.01) for the fluoroscopy-guided group (*p*=0.160). The changes in functional outcome after the procedure were compared similarly and are shown in Table 3. More subjective functional improvement was observed in the fluoroscopy-guided L-ESI (57, 60.6%) than in the blind L-ESI (47, 50.0%), although the difference was not statistically significant (*p*=0.16). Nine (9.6%) patients who experienced complications related to the procedure overall were in the blind L-ESI group and four (4.3%) patients were in the fluoroscopy-guided L-ESI group (*p*=0.27), as shown in Table 4. There were no significant adverse effects and sequelae recorded during and after L-ESIs were performed in either group of patients.

## 4. Discussion

This study was the first to review and compare outcomes and complications of blind and image-guided L-ESI techniques performed in the same individuals. Studying the effects of both procedures in the same patients gave us reliable information with which to compare postoperative efficacy, as the procedures were performed in exactly the same anatomical situation. Most previous studies have used heterogeneous patient groups with different diseases and variable anatomical conditions. Various cases of chronic low back pain could provide us with confounding data in comparisons of the postprocedural efficacy of both blind and image-guided L-ESIs [8, 9].

For patients with chronic low back pain in which imaging tests support that the etiology of the pain is the lumbar spine, L-ESI is one of the best treatment choices according to reliable evidence [10, 11]. However, inaccurate needle passage and steroid deposition into the subarachnoid space may lead to serious complications, including arachnoiditis, spinal cord injury, and postdural puncture headache [8]. Clearly, the procedure's accuracy and safety should be supported to enable successful pain relief. The fluoroscopy-guided procedure has become the primary technique used to enhance the technical accuracy of L-ESIs [11]. With this real-time imaging technique, the physician can see the depth and direction of the needle. It may not only increase safety, but also save the time needed to find the accurate interlaminar space and level of the target lumbar vertebra [8]. Growing evidence related to the fluoroscopy-guided ESI technique has shown that imaging is necessary to perform L-ESI [12, 13].

However, data suggest that the occupational exposure to radiation ultimately could cause irreversible tissue damage [4, 14, 15]. What is increasingly worrisome is that we have not yet reached a clear conclusion about the way and to what extent the occupational exposure to low level radiation affects the body [14]. We can assume such damage only indirectly with evidence supported by determining whether people who have been exposed to radiation continuously ultimately develop malignant cancer [14]. Thus, patients and, most importantly, physicians should be warned that the widespread use of fluoroscopy could have serious long-term outcomes.

Furthermore, although image-guided L-ESI is used more widely and commonly, it is still much more expensive than is blind L-ESI [1]. Arrangements for the operating room and fluoroscopy and recovery rooms are costly and time-consuming. Unlike previous reviews [1, 7], our findings demonstrated that blind and image-guided L-ESI had similar rates of incidence of complications and postprocedural functional outcomes, in that the differences between the two techniques in these measures were not statistically significant.

The limitations in this study included the following: (1) small sample size; (2) observer bias in reviewing the retrospective data; (3) heterogeneity of diagnoses, and (4) heterogeneity of various pain physicians who performed the procedures. In view of the widespread use of fluoroscopy, despite the fact that it provides more reliable data, the radiation risk and medical expense lead pain physicians to favor the older blind technique often. Larger sample sizes and more homogenous data are vital in future studies to clarify the remaining uncertainties about this subject.

## 5. Conclusion

In this study, we reached the conclusion that both blind and image-guided L-ESI techniques have similar efficacy with respect to postprocedural pain relief, functional improvement, and complication risk. Physicians should consider the advantages and disadvantages of the two techniques carefully and determine ways to individualize the procedures to decrease the risk of complications and improve positive outcomes after lumbar interlaminar epidural steroid injections.

## Figures and Tables

**Table 1:** Baseline characteristics of the study population.

Parameters	*N*=94
Age (years)	69.9 (67.7–72.0)
Gender (male/female)	64 (68.1)/30 (32.9)
Weight (kg)	62.2 (60.1–64.3)
Height (cm)	157.9 (156.2–159.6)
Body mass index (kg/m^2^)	25.0 (24.2–25.8)
Concurrent disease	
Hypertension/diabetes	50 (53.2)/24 (25.5)
Previous lumbar surgery	21 (22.3)
Spondylolisthesis	29 (30.9)
Diagnosis	
Lumbar spinal stenosis	
Central stenosis	53 (56.4)
Foraminal stenosis	40 (42.6)
Lumbar disc herniation	13 (13.8)
Lumbar facet joint syndrome	32 (34.0)
Postlumbar surgery syndrome	18 (18.0)
Compression fracture	37 (39.4)
Cancer (bone metastasis)	6 (6.4)

Data are expressed as mean (95% confidence interval) and number (%), appropriately.

**Table 2:** Differences in pain intensity between blind and fluoroscopy-guided interlaminar lumbar epidural block in the same individuals.

	Nonimage-guided	Fluoroscopy-guided	*p* value
Initial NRS	7.1 (6.8–7.4)	7.0 (6.7–7.4)	0.710
Postblock NRS	5.8 (5.3–6.3)	5.4 (4.8–5.9)	0.197
Differences in NRS	1.29 (0.94–1.65)	1.64 (1.28–2.01)	0.160
Follow-up period (weeks)	6.4 (5.5–7.3)	7.2 (6.2–8.3)	0.215

Data are expressed as mean (95% confidence interval).

**Table 3:** Comparison of subjective functional outcome changes between blind and fluoroscopy-guided lumbar interlaminar epidural block in the same individuals.

Functional outcome	Nonimage-guided	Fluoroscopy-guided
Better	47 (50.0%)	57 (60.6%)
No change or worse	47 (50.0%)	37 (39.4%)

*p* value was 0.155 by McNemar's test.

**Table 4:** Complications of blind and fluoroscopy-guided interlaminar epidural block in the same individuals.

	Nonimage-guided	Fluoroscopy-guided	*p* value^*∗*^
Complications	9 (9.6)	4 (4.3)	0.267
Failed block	1 (1.1)	0 (0.0)	
Difficult block	2 (2.1)	1 (1.1)	
Dural puncture	5 (5.3)	3 (3.2)	
Intravascular injection	1 (1.1)	0 (0.0)	

Data are expressed as number (%). ^*∗*^Overall *p* value was calculated by McNemar's test.

## Data Availability

The data used to support the findings of this study are available from the corresponding author upon request.
